# The Potential of Transcranial Direct Current Stimulation (tDCS) in Improving Quality of Life in Patients with Multiple Sclerosis: A Review and Discussion of Mechanisms of Action

**DOI:** 10.3390/jcm14020373

**Published:** 2025-01-09

**Authors:** James Chmiel, Donata Kurpas, Marta Stępień-Słodkowska

**Affiliations:** 1 Faculty of Physical Culture and Health, Institute of Physical Culture Sciences, University of Szczecin, Al. Piastów 40B Block 6, 71-065 Szczecin, Poland; 2 Department of Family and Pediatric Nursing, Faculty of Health Sciences, Wrocław Medical University, 51-618 Wrocław, Poland; donata.kurpas@umw.edu.pl

**Keywords:** tDCS, transcranial direct current stimulation, multiple sclerosis, quality of life, non-invasive brain stimulation, neurostimulation, neuromodulation

## Abstract

**Background/Objectives**: Multiple sclerosis (MS) is the most prevalent incurable nontraumatic neurological disability in young individuals. It causes numerous symptoms, including tingling, fatigue, muscle spasms, cognitive deficits, and neuropsychiatric disorders. This disease significantly worsens quality of life (QoL), and this dimension of general functioning provides valuable information about the effectiveness of treatment and well-being. There are psychological interventions that can improve QoL, but their number is limited. Therefore, searching for new methods that are as effective and safe as possible is ongoing. **Methods**: This review examines the potential effectiveness of transcranial direct current stimulation (tDCS) in improving the quality of life in patients with MS. Searches were conducted in the PubMed/Medline, Research Gate, and Cochrane databases. **Results**: The search yielded seven studies in which QoL was a primary or secondary outcome. Stimulation protocols displayed heterogeneity, especially concerning the choice of the stimulation site. Four studies demonstrated the effectiveness of tDCS in improving QoL, all of which (two) used anodal stimulation of the left DLPFC. Stimulation of the motor cortex has produced mixed results. The potential mechanisms of action of tDCS in improving QoL in MS are explained. These include improved synaptic plasticity, increased cerebral blood flow, salience network engagement through tDCS, and reduction of beta-amyloid deposition. The limitations are also detailed, and recommendations for future research are made. **Conclusions:** While the evidence is limited, tDCS has shown potential to improve QoL in MS patients in some studies. Prefrontal stimulation appears promising, and further research is recommended to explore this approach.

## 1. Introduction

Known as an immune-inflammatory disease, multiple sclerosis (MS) is the most prevalent incurable nontraumatic neurological disability in young individuals [1]. It causes unpredictable inflammatory bouts that can last anywhere from a few days to even months, causing damage to oligodendrocytes [2], myelin sheaths [3], and, to a lesser degree, nerve cells and axons [4]. There is little doubt that the immune system has a role in the pathogenesis of MS, directly or indirectly [5]. The majority of experts believe that primary autoimmunity is the pathogenic basis of MS [6]. Cerebrospinal fluid (CSF) usually contains evidence of acute and chronic inflammation, particularly during acute clinical episodes [7], as evidenced by the presence of distinct oligoclonal CSF IgG bands on gel electrophoresis and an increase in CSF IgG index [8], giving evidence of an elevation in the total CSF protein concentration [9] and an increase in the gamma-globulin (IgG) fraction in the CSF [10].

While the exact etiology of MS remains unknown [11], it is widely accepted that MS arises from a multifactorial interplay of genetic predisposition and environmental exposures [12,13]. Among the environmental risk factors, strong and consistent evidence suggests that Epstein-Barr virus (EBV) infection plays a particularly significant role in increasing the risk of MS, especially in genetically susceptible individuals, such as those carrying variations in the major histocompatibility complex [14,15]. In addition to EBV, other modifiable factors such as cigarette smoking [16], low vitamin D levels [17], and elevated BMI during adolescence [18] have also been associated with an increased risk of developing MS. While these factors may contribute to disease onset and progression, their impact is considered less direct than that of EBV infection [15,19,20].

It is essential that clinicians, in addition to protecting and prolonging life, consider human Quality of Life (QoL) [21] as a goal of comprehensive therapy [22]. Determining QoL involves detailing how a patient’s everyday life is affected by the illness and the treatment and completing the objective health status indicators [23]. QoL can also be assessed individually to assess the methods used and to create a treatment plan [24]. It can also be seen in a collective setting among patients with similar conditions, where a quality-of-life evaluation is a valuable tool to evaluate comprehensive programs or measure the impact of medication side effects [25]. Numerous studies have revealed a negative correlation between QoL and death or rehospitalization in individuals suffering from conditions such as respiratory disorders [26] and cardiovascular disease [27,28]. In patients with cancer [29,30], chronic renal disease [31], or following coronary bypass graft surgery [32], quality of life is also predictive of overall survival. A meta-analysis of approximately 1,200,000 people from the general population showed that a better quality of life is associated with lower mortality [33]. We do not have similar data for MS, but it seems likely that quality of life may be associated with an improved response to treatment and prognosis. Therefore, another importance of QoL testing in general clinical practice in patients with MS should be emphasized—it can potentially identify those with the highest risk of death.

Because MS is associated with many limitations, symptoms, dysfunctions, and unpredictable prognoses, this disease significantly worsens the quality of life of people affected [34,35,36,37,38,39]. Their reduced ability to function in daily life may cause this lower quality of life, especially if they need the assistance of caregivers, which might hinder social, professional, and family relationships [40]. Thus, it is just as important for physicians to evaluate the quality of life of MS patients as it is for the patients themselves to determine how their functional and mental states affect QoL. Taking steps to help patients feel better and to stabilize their physical and psychological health gives them a more positive outlook. Provider and patient perspectives of what constitutes a good quality of life during multiple sclerosis have shifted significantly due to the growing focus on health-related quality of life [41,42,43]. Upon examining website traffic in 2018, the National MS Society established a wellness research group to promote research to improve optimal health for people with MS [44]. The organization noticed individuals visiting the website seeking health and wellness ideas. These days, a person’s total quality of life while living with MS is what determines if they are living well with the disease rather than just the lack of relapses or the rate at which their impairment advances [45,46].

Interventions to improve the quality of life of MS patients mainly include psychological procedures. Among them, the effectiveness of mindfulness meditation [47,48,49], cognitive rehabilitation [50], and cognitive behavioral therapy [44] has been demonstrated. One meta-analysis of eight studies examining the effects of mindfulness on MS patients has demonstrated the effectiveness of meditation in improving well-being [49]. Although all these approaches are promising, there is a need to search for new therapeutic methods that are as effective and safe as possible and act directly on the brain, as MS is a neurological disease that significantly affects the patient’s mental sphere. One of the methods of influencing brain functioning, its reorganization, and neuroplasticity is transcranial direct current stimulation (tDCS). In recent years, this neuromodulation technique has been gaining much attention in neurological and psychiatric rehabilitation.

An anode and a cathode placed on the scalp are used in the tDCS technique to apply a low voltage and current (usually 1–2 mA) [51]. The electrical current is delivered via a battery-operated stimulator, with a portion of it reaching the brain. Stimulation produces a general pattern of increased cortical excitability beneath the anode and decreased excitability beneath the cathode (the reference/return electrode) [52] for up to ninety minutes following stimulation. In particular, tDCS regulates the rate at which neurons fire by altering the neuronal membrane potential in the specific brain region that is being stimulated [53]. Cathodal tDCS hyperpolarizes neurons and prevents them from firing; in contrast, anodal tDCS depolarizes neurons, allowing them to fire [54]. Similar to the processes seen in long-term potentiation (LTP) and long-term depression (LTD), the neuroplastic effects of tDCS are driven by the stimulation parameters and entail calcium-dependent modification of synaptic plasticity in n-methyl-D-aspartate (NMDA) glutamatergic neurons [55]. tDCS is a low-risk method with few adverse consequences [56]. tDCS is effective in alleviating the symptoms of many neurological and psychiatric diseases [57,58,59,60], even though the mechanisms of action are diverse and not fully understood. tDCS in the context of MS is also being intensively studied. Stimulation has been shown to benefit fatigue, pain, and motor symptoms [61,62,63,64,65,66,67]. However, there has yet to be a publication examining the collective impact of tDCS on the quality of life in people with MS, with the potential mechanisms of action of tDCS in this area unknown.

This review aims to investigate the possible uses of tDCS to enhance the quality of life for MS patients. Through regulating cortical excitability, tDCS can elicit alterations in behavior and facilitate neuronal reorganization. Since MS is linked to alterations in brain function, tDCS therapy may help to reorganize these abnormalities and directly or indirectly improve patient quality of life.

## 2. Methods

### 2.1. Data Sources and Search Strategy

For this review, J. Ch., D. K., and M. S.-S. performed an independent publication database search. The following keyword string was used: “transcranial direct current stimulation” OR “tDCS” AND “multiple sclerosis”. We considered publications in the PubMed/Medline, Research Gate, and Cochrane databases with an access date of February 2024 and publication dates ranging from January 2008 to February 2024.

### 2.2. Study Selection Criteria

Eligibility criteria comprised clinical trials conducted in English, published from 2008 to 2024, investigating the effects of tDCS on quality of life in multiple sclerosis as a primary or secondary outcome. The exclusion criteria encompassed articles not published in English and reviews.

### 2.3. Screening Process

The screening process was conducted in multiple stages to ensure the inclusion of relevant studies and the exclusion of those that did not meet the predefined criteria.

The initial screening involved a thorough examination of titles and abstracts by the reviewers (J. Ch., D.K., and M.S.-S.) independently.

#### 2.3.1. Title and Abstract Screening

Each reviewer independently assessed the titles and abstracts of retrieved records to identify studies that potentially met the inclusion criteria. During this stage, the screening criteria focused on the relevance of transcranial direct current stimulation and its effects on quality of life in multiple sclerosis.

#### 2.3.2. Full-Text Assessment

The selected articles underwent a comprehensive full-text assessment following the title and abstract screening. Reviewers examined the complete manuscripts to determine if they met the detailed eligibility criteria, emphasizing the inclusion of clinical trials conducted in English and published between January 2008 and February 2024.

## 3. Results

**Figure 1:**
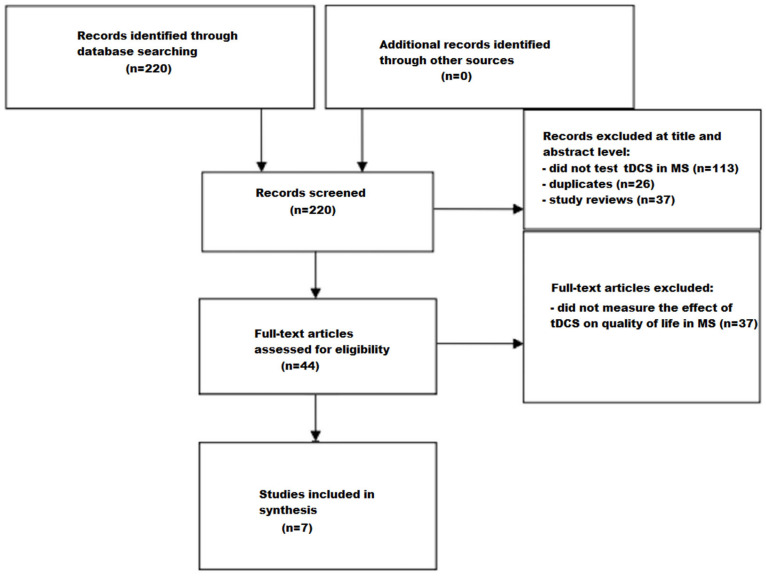
Flow chart depicting the different phases of the systematic review.

The screening process is illustrated in a flow chart (Figure 1). Through the search strategies carried out in the databases, 220 studies were identified. A total of 176 studies were excluded based on the evaluation of their titles and abstracts due to not testing tDCS in multiple sclerosis (*n* = 113), due to the removal of duplicates (*n* = 26), and due to the removal of study reviews (*n* = 37). Afterward, 44 studies were identified and underwent a comprehensive full-text assessment. Of these, 37 studies were excluded because they did not measure the effect of tDCS on quality of life in multiple sclerosis. Seven articles were deemed eligible for inclusion after a full reading of the texts.

### 3.1. Summary of Included Studies

The included studies are summarized in Table 1. The included studies [68,69,70,71,72,73,74] were published between 2010 and 2022. All studies except [71] used MSQoL-54 to measure quality of life. MSQoL-54 (Multiple Sclerosis Quality of Life-54) is a health-related quality of life (HRQoL) instrument specifically designed for individuals with MS. It is a comprehensive questionnaire that assesses the physical, mental, and emotional impacts of MS on a person’s daily life. By integrating both generic quality-of-life measures and MS-specific items, MSQoL-54 provides a holistic view of the disease’s effect on overall well-being. The questionnaire comprises 54 items divided into several components. The first component is the Physical Health Composite, with 14 scales that measure aspects such as physical function, role limitations due to physical problems, pain, energy and fatigue, health perceptions, social function, cognitive function, health distress, sexual function, overall quality of life, emotional well-being, role limitations due to emotional problems, change in health, and satisfaction with sexual function. The second component is the Mental Health Composite, which focuses on emotional well-being and role limitations due to emotional problems. Additionally, MSQoL-54 includes several MS-specific items that address issues particularly relevant to MS patients, such as bladder control, cognitive function, and fatigue [75].

The aim of the randomized double-blind sham-controlled parallel clinical trial by Mortezanejad et al. [68] was to investigate the effect of tDCS on fatigue and quality of life in patients with MS. Thirty-six volunteers were divided into three groups at random: primary motor a-tDCS (*n* = 12, mean age 33.35 years, 3 males, 11 females), sham a-tDCS (*n* = 12, mean age 32.50 years), and dorsolateral prefrontal cortex a-tDCS (*n* = 12, mean age 32.00 years, 1 male, 11 females). The study was open to people aged 18 to 50 years, without depression, and a relapse in the previous two months. Six sessions of a-tDCS (1.5 mA, 20 min) were administered to participants in the experimental groups over a period of two weeks, with three sessions each week. This intensity was selected based on the recommendations of Nitsche et al. regarding safe stimulation parameters for use in humans [76]. Quality-of-life assessments were conducted before, during, and four weeks following the sessions. Patients with multiple sclerosis had their quality of life evaluated using the Multiple Sclerosis Quality of Life questionnaire (MSQoL-54). Quality of life was the primary outcome. The active (anode) electrode was centered above the left primary motor cortex (C3) in the primary motor a-tDCS group. The left dorsolateral prefrontal cortex (DLPFC, F3) was covered by the active (anode) electrode in the dorsolateral prefrontal cortex a-tDCS group. The return (cathode) electrode for both groups was over the right contralateral supraorbital area. The mean MSQOL-54 score in the anodal tDCS to DLPFC group was 52.81, and in the anodal tDCS to M1 group, it was 51.68.

The aim of the randomized double-blind sham-controlled study by Mori et al. [69] was to investigate the effect of anodal tDCS stimulation of the motor cortex on chronic pain, quality of life, anxiety, and depression in 19 patients (mean age 44.8 years) with relapsing-remitting MS in the remitting phase. Nine patients were placed in the group receiving sham tDCS, and ten patients were randomly assigned to receive active tDCS (5 males, 5 females). MSQoL-54 was used to measure quality of life. Quality of life was a secondary outcome. Assessments were conducted at baseline, right after treatment concluded, and once a week for the next three weeks. The painful somatic area was contralateral to the anode electrode, positioned over the C3 or C4 location. The supraorbital region, which was contralateral to the motor cortex, was activated by the cathode electrode. A continuous current of 2 mA intensity was administered once daily for 20 min for five days in a row.

In the randomized controlled single-blind study by Young et al. [70], the aim was to investigate the effect of tDCS on chronic pain, depression, anxiety, and quality of life in patients with MS. Thirty participants were included in the trial; fifteen were randomly assigned to an active group (mean age 51.2 years, 11 females, 4 males) and the remaining fifteen to a sham group (mean age 49.87 years). Most patients had the relapsing-remitting type of MS. For five days in a row, a current of 2 mA intensity was used with the following protocol: 10 min of stimulation, 25 min without stimulation, and another 10 min of stimulation. Quality of life was assessed weekly up to four weeks following the five-day treatment of a-tDCS. Quality of life was measured using MSQoL-54. Quality of life was a secondary outcome. The side with the pain was identified by applying the anodal electrode to the C3 or C4 contralateral; if both sides were affected, the side with a higher pain threshold was chosen. The supraorbital region, which was contralateral to the activated motor cortex, was covered by the cathode electrode.

In the cross-over study by Muñoz-Paredes et al. [71], the aim was to evaluate the efficacy of two distinct programs—one centered on tDCS and the other on the impact of physical activity on fatigue and quality of life in MS patients. Twelve individuals (mean age 48.08 years) with progressive secondary MS (*n* = 5) and relapsing-remitting MS (*n* = 7) took part in the study. Quality of life was evaluated both before and after the intervention. The Multiple Sclerosis International Quality of Life (MusiQoL) questionnaire was administered to evaluate quality of life. Quality of life was the primary outcome. Four weeks were dedicated to the exercise regimen and tDCS, followed by a five-month hiatus. The anode was placed over the left DLPFC (F3), and the cathode was placed over the right supraorbital cortex. Over the course of four weeks, ten sessions of twenty minutes each were applied. The current was 2 mA. There was no control group condition. The mean MusiQoL score in the tDCS group before treatment was 69.658. This score was obtained by adding all 10 MusiQoL subscales and then dividing by 10.

A double-blind randomized controlled trial by Rahimibarghani et al. [72] enrolled thirty-nine participants in the study: 21 in the active group (mean age 40.0 years, 13 females, 8 males) and 18 in the control group (mean age 39.8 years). In the tDCS group, 12 individuals (57.1%) had secondary progressive MS, 3 (14.3%) had relapsing-remitting MS, and 6 (28.6%) had primary progressive MS. Participants were assigned randomly to exercise on a stationary bike in conjunction with anodal tDCS or with a sham tDCS protocol. MSQoL-54 was used to measure quality of life. Quality of life was a secondary outcome. Twelve tDCS sessions were performed over six weeks. The sessions lasted 20 min, and the current intensity was 1.5 mA. The motor cortex region (C3) was the location of the anode electrode. The placement of the cathode was extracephalic on the other shoulder. In the tDCS group, the MSQoL-54 physical health composite score before treatment was 50.16, while the MSQoL-54 mental health composite score was 44.40.

The aim of the randomized, double-blind, sham-controlled study by Mori et al. [73] was to check the effect of tDCS on tactile sensory deficit and quality of life in relapsing-remitting MS patients. 20 participants took part in the study (10 in the active tDCS group (mean age 42.3 years, 4 males, 6 females) and 10 in the sham tDCS group (mean age 40.0 years). Quality of life was measured using MSQoL-54. Quality of life was a secondary outcome. The anode electrode was applied to the scalp 2 cm posterior to the 10–20 EEG system’s C3 or C4 position, contralateral to the hypoesthetic upper limb, to stimulate the somatosensory cortex (S1). The supraorbital region, opposite to the activated sensory cortex, was covered by the cathode electrode. 5 tDCS sessions were used with an intensity of 2 mA and a duration of 20 min. This intensity was selected based on previous studies demonstrating its effectiveness in treating pain. Outcomes were measured immediately after the intervention and at 1, 2, and 4 weeks after the intervention. No numerical data were provided for the results of quality-of-life measures before intervention.

The purpose of the randomized clinical trial study of Mohammadkhanbeigi et al. [74] was to investigate the effect of core stability exercises and tDCS on balance, walking capacity, and quality of life in patients with MS. For three weeks, participants in the core stability training group engaged in core stability exercises, while those in the sham and anodal tDCS groups underwent five sessions of tDCS. The study included people in a stable condition, without relapse. The type of MS the people included in the study had was not specified. 29 patients participated in the study (10 in the core stability training group (mean age 40.20 years), 9 in the anodal tDCS group (mean age 37.44 years, only females), and 10 in the sham tDCS group (mean age 37.70 years). A single session lasted 20 min with a current intensity of 2 mA. The MSQoL-54 measured quality of life. Quality of life was the primary outcome. According to the international 10–20 EEG System, the anode electrode was positioned over the M1 cortex, 10–20% anterior to CZ in the midline, and the cathode electrode was situated over the left supraorbital region.

**Table 1:** Summary of main findings from articles included in the review.

Author, Citation	Population	Test Used	Intervention	Anodal Stimulation Site	Current Intensity	Duration (Min)	Main Findings in Treatment Group
Mortezanejad et al. [68]	36 participants in 3 groups (primary motor: *n* = 12, sham: *n* = 12, DLPFC: *n* = 12)	MSQoL-54	6 sessions over 2 weeks (3 sessions/week)	Primary motor: C3, DLPFC: F3	1.5 mA	20	Both the primary motor and dorsolateral prefrontal cortex groups experienced a significant increase in quality of life immediately following the intervention (mean = 52.81 ± 3.10 before the DLPFC a-tDCS intervention; mean = 64.23 ± 1.80 after the DLPFC a-tDCS; mean = 51.68 ± 3.60 before the primary motor a-tDCS intervention; mean = 62.91 ± 2.95 after the primary motor a-tDCS intervention). But only in the dorsolateral prefrontal cortex group—mean = 62.85 ± 1.78 in that group; mean = 55.20 ± 2.81 in the primary motor a-tDCS group—did the improvement in quality of life hold up against the sham group one month after the intervention (*p* < 0.02). Furthermore, one month after the intervention, the primary motor a-tDCS group’s quality of life reverted to baseline (*p* < 0.01).
Mori et al. [69]	19 participants (active: *n* = 10, sham: *n* = 9)	MSQoL-54	5 sessions over 1 week	Somatic area contralateral to pain (C3/C4)	2 mA	20	Repetitive measures ANOVA was used in the study [69] to analyze MSQOL-54 scores with time as within-subjects (baseline, post one week, post two weeks, post three weeks, and post four weeks) and treatment group as between subjects (active vs. sham tDCS). The results indicated that time (F = 3.04, *p* < 0.05) and group × time (F = 3.18; *p* < 0.05) were significant effects. Following the first week of stimulation, Duncan’s correction revealed a considerable difference that persisted until the final evaluation (all *p* < 0.05).
Young et al. [70]	30 participants (active: *n* = 15, sham: *n* = 15)	MSQoL-54	5 sessions over 1 week	Side of pain contralateral (C3/C4)	2 mA	20 (10 min of stimulation, 25 min of nonstimulation, and another 10 min of stimulation).	No improvement in the quality of life.
Muñoz-Paredes et al. [71]	12 participants	MusiQoL	10 sessions over 4 weeks	DLPFC (F3)	2 mA	20	Significant improvements and clinical alterations were observed (*p* = 0.015) (g = 0.646) after the application of tDCS. Furthermore, statistical analysis reveals that the subscales of activities ADL (*p* = 0.037) (g = 0.465), PWB (*p* = 0.004) (g = 0.727), and COP (*p* = 0.18) (g = 0.376) have undergone substantial modifications following the administration of the tDCS.
Rahimibarghani et al. [72]	39 participants (active: *n* = 21, control: *n* = 18)	MSQoL-54	12 sessions over 6 weeks	C3	1.5 mA	20	Both interventions improved the quality-of-life index, both immediately after the intervention and after a four-week observation period.
Mori et al. [73]	20 participants (active: *n* = 10, sham: *n* = 10)	MSQoL-54	5 sessions over 1 week	Somatosensory cortex (S1, C3/C4)	2 mA	20 min	No improvement in quality of life was demonstrated at any time point after the intervention.
Mohammadkhanbeigi et al. [74]	29 participants (active: *n* = 9, sham: *n* = 10, core stability: *n* = 10)	MSQoL-54	5 sessions over 1 week	Motor cortex (M1, 10–20% anterior to Cz)	2 mA	20 min	No improvement in quality of life was demonstrated.

Abbreviations: A-tDCS-anodal tDCS, DLPFC-dorsolateral prefrontal cortex, MSQoL-54-Multiple Sclerosis Quality of Life questionnaire, mA-miliamperes, MusiQoL- Multiple Sclerosis International Quality of Life.

### 3.2. Impact on Quality of Life

A study [68] found that both the primary motor and dorsolateral prefrontal cortex groups experienced a significant increase in quality of life immediately following the intervention (mean = 52.81 ± 3.10 before the dorsolateral prefrontal a-tDCS intervention; mean = 64.23 ± 1.80 after the dorsolateral prefrontal a-tDCS; mean = 51.68 ± 3.60 before the primary motor a-tDCS intervention; mean = 62.91 ± 2.95 after the primary motor a-tDCS intervention). But only in the dorsolateral prefrontal cortex group—mean = 62.85 ± 1.78 in that group; mean = 55.20 ± 2.81 in the primary motor a-tDCS group—did the improvement in quality of life hold up against the sham group one month after the intervention (*p* < 0.02). Furthermore, one month after the intervention, the primary motor a-tDCS group’s quality of life reverted to baseline (*p* < 0.01).

Repetitive measures ANOVA was used in the study [69] to analyze MSQoL-54 scores with time as within-subjects (baseline, post one week, post two weeks, post three weeks, and post four weeks) and treatment group as between subjects (active vs. sham tDCS). The results indicated that time (F = 3.04, *p* < 0.05) and group × time (F = 3.18; *p* < 0.05) were significant effects. Following the first week of stimulation, Duncan’s correction revealed a considerable difference that persisted until the final evaluation (all *p* < 0.05). No specific numerical data were given for the outcome. From the graph, it can be seen that the MsQoL-54 scores before treatment were around 53, and after the first week of treatment, they were around 75. The graph also shows that the scores were maintained at weeks 2, 3, and 4 of treatment.

A study [70] showed non-significant improvement in the quality of life. More specifically, the MSQoL54 Physical subscore improved in the active tDCS group from 47.9 to 52.5, while the MSQoL54 Mental subscore improved from 68.3 to 70.2. This improvement was greater than in the sham tDCS group, where scores improved by 0.8 and 0.1 points.

In the study [71], significant improvements and clinical alterations were observed (*p* = 0.015) (g = 0.646) after the application of tDCS. Furthermore, statistical analysis reveals that the subscales of activities ADL (*p* = 0.037) (g = 0.465), PWB (*p* = 0.004) (g = 0.727), and COP (*p* = 0.18) (g = 0.376) had undergone substantial modifications following the administration of the tDCS. The mean post-treatment score in the tDCS group was 75.426.

A study [72] showed that both interventions improved the quality-of-life index, both immediately after the intervention and after a four-week observation period. In the tDCS group, the MSQoL-54 physical health composite score after treatment was 53.99, and after 4 weeks it was 50.00. The MSQoL-54 mental health composite score after treatment was 45.72, and after four weeks it was 44.84.

In the study [73], no improvement in quality of life was demonstrated at any time point after the intervention. No numerical data were provided regarding the outcomes in quality-of-life measures after the intervention.

In the study [74], no improvement in quality of life was demonstrated (the result dropped from 63.15 to 62.15).

Because the MSQoL54 is the most commonly used tool to measure the quality of life in people with MS, and this scale consists of several components, the exact scores on the MSQoL54 in individual studies are presented in Table 2.

### 3.3. Safety

tDCS did not cause any side effects in the included studies. The comments that did appear were mainly about tingling.

### 3.4. Risk of Bias

To assess the risk of bias in the included studies, a standardized approach, such as the Cochrane Risk of Bias Tool (RoB 2) for randomized controlled trials, was applied. The following provides a continuous narrative summary of the findings across the key domains of the tool.

The first domain, bias arising from the randomization process, examines whether participants were adequately randomized and if allocation concealment was ensured. Most studies, including those by Mortezanejad et al. [68] and Mori et al. [69], employed randomization procedures, such as computer-generated randomization lists. However, while these studies claimed to be double-blind, detailed descriptions of allocation concealment procedures were often missing, making it challenging to fully assess their robustness. Therefore, the risk of bias in this domain is generally categorized as low to unclear, depending on the completeness of the descriptions.

The second domain, bias due to deviations from intended interventions, evaluates the adequacy of blinding and adherence to intervention protocols. Many studies ensured blinding by using sham stimulation setups, such as applying electrodes without delivering current in the sham groups [68,69,73]. However, blinding might not have been fully effective in some cases due to the physical sensations associated with active tDCS, as noted in Young et al. [70]. The overall risk of bias for this domain is considered low, though it could be moderate in studies where incomplete blinding might have influenced the results.

The third domain, bias due to missing outcome data, assesses whether dropout rates were reported and appropriately managed. Most studies provided clear information about dropout rates and their reasons. For instance, Muñoz-Paredes et al. [71] and Rahimibarghani et al. [72] explicitly reported dropout rates and employed intention-to-treat analyses to account for missing data. This approach reduces the risk of bias in this domain, which is generally rated as low across the included studies.

The fourth domain, bias in the measurement of outcomes, considers whether outcome assessors were blinded and if validated tools were used. Outcome measurement in most studies adhered to rigorous standards, with validated instruments such as the MSQoL-54 scale and Fatigue Severity Scale employed to evaluate the impact of interventions [68,69,71]. Blinding of outcome assessors was consistently reported, further minimizing potential bias. Consequently, the risk in this domain is categorized as low for the majority of studies.

The fifth domain, bias in the selection of the reported results, examines whether pre-specified outcomes were reported and if selective reporting was evident. Most studies followed pre-registered protocols or adhered to well-established methods, as seen in Mortezanejad et al. [68] and Young et al. [70]. However, some studies, such as Muñoz-Paredes et al. [71], lacked clarity on pre-specified outcomes, introducing the potential for reporting bias. This domain is rated as low to unclear, depending on the level of detail provided.

## 4. Discussion

As a chronic neurological condition known for its wide variety of symptoms, multiple sclerosis can have a major negative influence on a patient’s quality of life. A promising therapeutic method for the management of MS symptoms, such as fatigue, chronic pain, and motor dysfunction, is tDCS. Studies have also examined the impact of tDCS on the quality of life of MS patients, as this aspect is essential in measuring the treatment effectiveness of a given intervention. This discussion focuses on the impact of tDCS on the quality of life in MS, as assessed in various randomized controlled trials and studies. Moreover, the following discussion explains the potential mechanisms of action of tDCS in improving quality of life during MS.

### 4.1. Effectiveness of tDCS on Quality of Life in MS

The findings from the included studies provide a mixed picture of the effectiveness of tDCS in improving the quality of life in patients with MS. Mortezanejad et al. [68] reported significant improvements immediately following tDCS interventions targeting both the primary motor cortex (M1) and the dorsolateral prefrontal cortex (DLPFC), with sustained benefits observed only in the DLPFC group at a one-month follow-up. Similarly, improvements in quality of life were noted after M1 stimulation in the study [69] and after left DLPFC stimulation in the study [71]. In contrast, Young et al. [70] observed an improvement in the M1 group that did not reach statistical significance, while Mori et al. [73] and Mohammadkhanbeigi et al. [74] reported no improvements. The available evidence suggests consistently positive results when anodal stimulation of the left DLPFC is used; however, more studies are needed to draw definitive conclusions.

Differences in outcomes may stem from several factors. First, the number of tDCS sessions varied: studies showing no effect on quality of life [70,73,74] applied only five sessions, while those reporting benefits included protocols with five [69], six [68], ten [71], or twelve [72] sessions. Second, most studies—except [72], which lacked a sham control—were randomized controlled trials of reasonable quality but involved relatively small sample sizes, limiting the power to detect statistically significant changes. Third, session duration and current intensity (1.5–2 mA) varied between the studies, complicating direct comparisons. Fourth, differences in MS subtypes across trials and the lack of specification in some studies introduced additional heterogeneity. Finally, while tDCS shows promise for improving quality of life, it remains an emerging intervention compared to established therapies. Combining tDCS with other modalities—such as CBT, mindfulness, or physical therapy—may yield additive or synergistic benefits.

From a clinical perspective, these findings underscore the potential of tDCS as an adjunct therapy for MS-related symptoms and overall quality of life, particularly when targeting the left DLPFC. Multi-session protocols—typically involving at least five sessions—are associated with more consistent benefits compared to shorter protocols. Given the DLPFC’s role in emotional regulation and executive function, stimulation at this site may yield broader impacts on mood, cognition, and coping, which collectively influence well-being. Although optimal protocols remain undefined, intensities of 1.5–2 mA administered for 20 min per session are commonly used and generally safe, with good patient tolerability. Clinicians should monitor side effects (e.g., headache, tingling, or skin irritation) and ensure correct electrode placement to maximize safety and efficacy. While larger, methodologically rigorous trials are needed to confirm the benefits of tDCS for improving quality of life in MS, current evidence suggests it holds promise when delivered in repeated sessions, particularly over the left DLPFC, and when combined with other therapeutic strategies.

The available data suggest that tDCS intensities of 1.5–2 mA delivered over 20-min sessions are safe and potentially beneficial for improving quality of life in patients with MS, though the specific number of sessions and the targeted cortical region can significantly influence outcomes. The use of intensities of 1.5 and 2 mA is standard in tDCS applications for MS, as supported by review studies. These intensities are known to induce neuroplastic changes that underlie the effects of stimulation. Protocols involving at least five sessions, and often more, appear more likely to produce lasting benefits, while the choice of stimulation site (most notably the left DLPFC) may be particularly advantageous when emotional regulation or cognitive factors contribute to a patient’s reduced well-being. While M1 stimulation can alleviate physical symptoms such as chronic pain, evidence regarding its direct impact on overall quality of life remains mixed. Scheduling daily or every-other-day sessions, with follow-up evaluations at regular intervals, is important for capturing both short-term gains and the durability of effects. Given the small sample sizes in many studies, future research with larger cohorts and standardized protocols is needed to refine the understanding of how current intensity, electrode placement, and session frequency can be optimized for different MS subtypes.

The impact of gender on quality of life is not extensively analyzed in the provided studies. However, gender-related data is available in participant demographics, which could inform future discussions or analyses. For instance, a study [68] reports the gender composition in its groups: 1 male and 12 females in the DLPFC a-tDCS group, 3 males and 11 females in the M1 a-tDCS group, and 1 male and 7 females in the sham group. Similar demographic data are present in other studies but lack specific analysis of gender as a factor influencing quality of life. While none of the studies directly examine how gender affects outcomes, general considerations suggest potential differences. Women, who are more frequently affected by MS, may experience variations in symptomatology or treatment response compared to men, potentially influencing quality-of-life improvements. The predominance of female participants reflects the higher prevalence of MS among women, potentially limiting the generalizability of findings among male patients.

The quality of the included studies does not seem to have influenced the discrepancy in the results. Studies [69,70,71,73,74] are of good quality as they are RCTs but have a low number of patients. They also contained a control group. The study [72] is of low quality as it is a pilot study with a low number of patients and lacks a sham control group.

It is not possible to determine the influence of the type of MS on the discrepancy in the results. The patients had different types of MS. Some studies did not report the type of MS.

### 4.2. Mechanisms of Action of tDCS in Improving Quality of Life in MS Patients

#### 4.2.1. tDCS May Improve Synaptic Plasticity

The nervous system’s pervasive characteristic of synaptic plasticity enables neurons to interact and alter their connections in response to previous experiences [77]. There is evidence that synaptic plasticity is impaired in people with MS, and interventions that enhance this plasticity can improve functioning and quality of life [78]. Using tDCS to target the compromised synaptic plasticity may be seen as a highly effective method of treating MS. During and after stimulation, tDCS may cause polarization of the membrane and polarity-specific changes in cortical excitability and may affect the conductance of sodium and calcium channels. These alterations are linked to neuroplastic changes and offer a potential explanation for enduring after-effects [55]. Neuroplastic alterations linked to NMDA receptors were necessary for the long-lasting effects of tDCS [55]. One of the main physiological bases of tDCS is altered intracellular Ca^2+^ uptake and NMDA receptor function [79]. Cathodal stimulation decreases cortical excitability, whereas anodal tDCS increases cortical activity. The postsynaptic neuron’s intracellular Ca^2+^ dramatically increased when anodal tDCS activated the NMDA receptor [80]. The degree of NMDA receptor activation determines how tDCS affects synaptic plasticity [80]. LTD-like alterations will result from a little increase in intracellular Ca^2+^ in the postsynaptic neuron [80]. Conversely, a slight increase in Ca^2+^ causes no change in synaptic plasticity, but a more significant increase results in LTP-like alterations [80]. tDCS may therefore regulate overactivated LTP in MS (which leads to excitotoxicity [81], loss of the ability to induce synaptic plasticity [78], and cognitive deficits [82]), alleviating the symptoms of the disease, bringing indirectly or directly an improvement in the quality of life.

#### 4.2.2. tDCS May Increase Cerebral Blood Flow

In patients with relapsing-remitting or secondary progressive multiple sclerosis, cerebral blood flow is decreased in the grey matter [83,84,85] and white matter [86,87] regions of the brain. This common clinical characteristic of multiple sclerosis, typically referred to as chronic cerebral hypoperfusion, lowers brain oxygenation and causes hypoxia [88]. Enhancing cerebral blood flow may mitigate the impairments observed in human patients with multiple sclerosis [89], thus affecting quality of life. tDCS has been shown to enhance cerebral blood flow in healthy subjects. However, after the tDCS was discontinued, the cerebral blood flow went back to normal. Furthermore, there was a linear correlation between the intensity of tDCS and the change in cerebral blood flow, indicating that tDCS regulation of cerebral blood flow is intensity-dependent. Contrary to anodal tDCS, cathodal tDCS caused a persistent decline in cerebral blood flow relative to baseline and lowered cerebral blood flow during stimulation [90]. Similar results were shown in a study on rats, with the difference that the effects of tDCS regulating cerebral blood flow lasted longer than just during stimulation [91]. In MS patients, one study employing positron emission tomography (PET) to evaluate rCBF in individuals with relapsing-remitting MS found no immediate changes in either regional or global blood flow following tDCS over the primary motor cortex at intensities ranging from 1 to 4 mA [92]. Another investigation highlighted variability in blood flow responses to tDCS, with some subjects showing expected changes in rCBF at specific intensities and stimulation sites (e.g., dorsolateral prefrontal cortex or primary motor cortex), while others exhibited unexpected or no significant alterations [93]. However, these studies were limited to case series with small patient cohorts, and the findings should be interpreted cautiously. Future research should explore whether the effects of tDCS increasing cerebral blood flow in MS patients contribute to the reduction of symptoms and, at the same time, improve the quality of life.

#### 4.2.3. Salience Network Engagement Through tDCS

Non-invasive brain stimulation like tDCS, if focusing on strengthening cognitive control of attention by increasing the excitability of appropriate brain areas included in the salience network (primarily the prefrontal cortex and the connected dorsal anterior cingulate cortex (dAAC)), may be another mechanism that improves quality of life in MS patients if well-being (and quality of life) is associated with the ability to integrate personally relevant and significant information via the salience and default mode networks [94]. It has been shown that tDCS, applied prefrontally, has network effects on various brain areas, including the salience network [95] and the default mode network [96]. Additionally, anodal stimulation of the left prefrontal cortex through tDCS may be an effective form of improving the quality of life, as EEG and fMRI studies have shown that higher levels of well-being are associated with greater activity of the left prefrontal cortex [94].

#### 4.2.4. Reduction of Beta-Amyloid Deposition

While beta-amyloid accumulation is a hallmark of Alzheimer’s disease (AD) [97], new research indicates that disrupted Aβ homeostasis may be a critical element connecting inflammation, synaptic plasticity, and cognitive impairment in multiple sclerosis [98]. Cognitive deficits in MS negatively contribute to the decline in quality of life in this group of patients [99,100]. One tDCS study has shown that tDCS can decrease amyloid plaque development. For instance, 10-day tDCS was administered to the mice’s skulls above the frontal cortex in an early-stage AD mouse model. While recognition memory did not increase in the mice who underwent tDCS, spatial learning and memory did dramatically improve. Notably, the tDCS-treated group exhibited a considerable reduction in Aβ42 deposition, offering direct evidence for mitigating the pathogenic alteration associated with AD [101]. Further research is required to confirm the role of amyloid beta in MS and to test whether tDCS can reduce its deposition in MS.

## 5. Research Limitations and Future Directions

Although the studies reviewed offer insightful information, it is important to recognize that there are a number of limitations that affect how the findings should be interpreted and identify areas for further research.

### 5.1. Small Sample Sizes and Heterogeneity of Patient Populations

Small sample sizes and heterogeneous patient demographics remain significant limitations in the generalizability and reliability of tDCS findings in MS research. Small trials often lack the statistical power needed to detect clinically meaningful effects on quality of life. Additionally, factors such as disease severity, duration, and comorbidities introduce variability that may obscure the therapeutic potential of tDCS. Variations in disability levels and disease subtypes (e.g., progressive vs. relapsing-remitting MS) further complicate the extrapolation of findings to broader MS populations. To enhance the reliability and applicability of results, future research should focus on larger, more homogenous cohorts. Multicenter studies with standardized inclusion criteria and outcome measures would provide more robust evidence regarding the effectiveness of tDCS in enhancing the quality of life for MS patients. Furthermore, subgroup analyses based on key patient characteristics (e.g., disease subtype, baseline impairment) could help identify predictors of treatment response and facilitate personalized intervention strategies. Addressing these methodological challenges will be critical to solidifying the role of tDCS in the management of MS-related quality-of-life issues. Additional key considerations are outlined in the following subsections.

### 5.2. Variation in Stimulation Protocols

The lack of consistency in tDCS protocols in MS research—including differences in electrode placement, current intensity, session length, and frequency—complicates cross-study comparisons and hampers the ability to draw definitive conclusions about the most effective approaches to improve patients’ quality of life. These parameters have a significant influence on cortical excitability and, consequently, therapeutic outcomes. To address this variability, future research should prioritize the standardization of stimulation protocols to improve reproducibility and enable meaningful meta-analyses. Establishing consensus on critical parameters, guided by preclinical and early clinical data, would help optimize tDCS interventions. Moreover, rigorous dose-response investigations are essential for identifying the most effective stimulation strategies for improving the quality of life in MS.

### 5.3. Short-Term Follow-Up

One of the most common limitations in tDCS research for multiple sclerosis is the lack of extended follow-up evaluations. While immediate post-intervention assessments provide insight into short-term effects, they offer limited information about the sustainability and long-term impact of tDCS on quality of life. Many studies include follow-up periods ranging from only one to four weeks, potentially missing delayed or cumulative effects. Given the chronic and progressive nature of MS, it is crucial to determine whether the benefits of tDCS persist beyond the initial treatment phase. Future research should incorporate extended follow-ups, ideally spanning several months to years, to provide a clearer understanding of the durability of tDCS-induced gains. Additionally, the potential utility of booster sessions or maintenance interventions should be investigated to optimize the long-term efficacy of tDCS as a strategy for improving the quality of life in MS patients.

### 5.4. Lack of Consensus on Outcome Measures

A lack of standardized outcome measures poses a significant challenge for interpreting and comparing findings from tDCS trials in MS. While some studies employ validated tools like the MSQoL-54, others use a variety of scales and endpoints (pain, mood, functional disability), with differing evaluation timelines. This diversity limits the ability to synthesize data and draw consistent conclusions about the impact of tDCS on quality of life. Moreover, current quality-of-life measures often lack defined normative standards, are geared toward general populations rather than MS-specific concerns, and fail to account for phenomena such as disease adaptation or response shifts [39]. To address these challenges, future tDCS research should adopt a core set of validated outcome measures for both quality of life and relevant symptom domains while incorporating standardized evaluation procedures and follow-up intervals. These steps would facilitate more robust data comparisons and strengthen the evidence for tDCS as an effective therapeutic intervention to improve the quality of life in MS patients.

### 5.5. Taking into Account Factors Influencing the Quality of Life of Patients with MS

#### 5.5.1. Co-Occurrence of Depression

Depression is a common disease comorbid with MS and significantly contributes to the decline in quality of life in this group of patients [102,103,104,105,106,107]. Men suffer from more severe symptoms of depression in MS than women [108,109], and their occurrence is influenced by a range of factors. For example, the severity of depression symptoms is correlated with education level; those who have completed college tend to have less depressive symptoms [110,111]. Therefore, in future studies on the impact of tDCS on the quality of life of patients with MS, it is important to measure depression, consider the impact of depression scores on quality-of-life scores, and observe the correlation between the occurrence and improvement of both indicators. The impact of the depression treatment used during the study on improving the quality of life should also be considered because research revealed that following effective therapy for depression, patients evaluated their quality of life higher [112,113].

#### 5.5.2. Duration of the Disease

Another factor that reduces QoL is the duration of the MS. It mostly impacts the assessment of the physical realm. Patient quality of life declines as their sickness worsens and they become more reliant on others. However, this does not apply to the mental sphere. According to the research by Pashazadeh et al. [114], there was a positive correlation between the length of the disease and mental HRQoL in MS patients, with the mental HRQoL score rising by 1.08 for every year of illness. This can be the result of patients’ optimism and acceptance of the changes the illness has brought about in their lives. Studies that have been reviewed by Pashazadeh et al. [114] have demonstrated an inverse relationship between the length of the disease and physical HRQoL, with a physical HRQoL score decreasing by 0.23 for every year that the condition lasts. This may be the result of the disease’s physiological effects on a person’s body over time, which lead to a decline in physical capabilities. Therefore, it is necessary for future studies on the impact of tDCS on quality of life in people with MS to consider the duration of the disease on the quality of life of patients, as well as the separate impact on physical and mental areas.

#### 5.5.3. Social Connections

Numerous studies show a relationship between the presence of friends and loved ones and the QoL assessment [102,104,115,116]. They boost the quality-of-life evaluation by offering a safety net and a sense of security, as well as making it easier to adjust to the restrictions of the illness. It may therefore be valuable to measure levels of social connections in MS patients and create homogeneous groups to exclude differential effects of this parameter on tDCS outcomes.

#### 5.5.4. Place of Residence

How a patient’s place of residence influences quality of life assessment is not controversial. Research by Yamout et al. [117], Buchanan et al. [118], and Jamroz-Wiśniewska et al. [119] revealed that people who live in cities had better quality of life. This is most likely because urban residents have easier access to physicians with a variety of specializations, cutting-edge medications, and other therapeutic modalities; consequently, they are more likely to get the support they need in order to get through challenges [102]. MS patients can participate in a variety of activities and join support groups in towns and cities; transportation is frequently provided. Each of these exercises supports patients in continuing to be as active and productive as they can be [102]. Therefore, we suggest monitoring this parameter in tDCS research to determine whether it may have an impact on improving quality of life results and in creating homogeneous study groups based on place of residence.

#### 5.5.5. Gender

According to the study by Kołtuniuk et al. [102], the impact of multiple sclerosis on the quality of life of women is much worse. Evaluating the quality of life in the areas of mental health (PWB) and disease symptoms (SPT) was correlated with the female gender. Men scored these two areas substantially higher than women on the MusiQoL scale. According to a study by Natarajan et al. [120], females score worse on the MusiQoL questionnaires in psychological well-being and coping than males. According to Miller and Dishon [121], women with MS experience more severe symptoms than males do, which have a big influence on everyday functioning and general well-being. The reviewed studies did not extensively examine the specific impact of gender on quality-of-life outcomes in patients undergoing tDCS treatments for MS. However, some insights can be inferred based on the demographic data and existing knowledge of MS. Most studies predominantly included female participants, consistent with the higher prevalence of MS among women. For instance, Mortezanejad et al. [68] reported 11 females and 1 male in one group, while Rahimibarghani et al. [72] included 13 females and 8 males in their active tDCS group. This gender imbalance may hinder the ability to discern whether tDCS affects males and females differently. While none of the studies directly analyzed gender-related differences, existing literature suggests that men and women may experience variations in symptoms, such as pain, fatigue, and cognitive challenges, which could influence their perceived quality of life. For example, men with MS often report higher levels of depression, which may affect their responsiveness to interventions such as tDCS. To address these gaps, future studies should stratify participants by gender to analyze outcomes accordingly to determine whether tDCS efficacy varies between men and women. This approach could help identify whether both genders derive similar benefits from tDSC or if tailored protocols are needed to optimize treatment for each group. Incorporating gender as a covariate in statistical models could provide more nuanced insights while subgroup analyses might uncover differences in response to specific parameters, such as session frequency or the targeted cortical regions. While the current evidence offers no definitive conclusions regarding the role of gender in tDCS-mediated quality-of-life improvements, future research should prioritize gender-specific analyses to enhance understanding and inform the development of personalized treatment approaches.

#### 5.5.6. Financial and Employment Status

Another important aspect influencing the patient’s quality of life is their employment and financial status. Patients rate their health and capacity for independent living higher when their financial circumstances are better. Patients have a higher quality of life when their money enables them to live comfortably [102,122]. Higher earners gave their social and physical quality of life higher ratings. Furthermore, affluent patients have better access to a variety of medical professionals and treatment modalities, which enhances both their state of health and the range of independent activities they can engage in [102]. Additionally, it was discovered that the main sociodemographic factor affecting QoL was employment [123]. Numerous research [124,125] showed a correlation between unemployment and a lower quality of life. A positive link was observed by others between QoL in MS, high employment status [126], job match and job satisfaction [127], and jobs suited to a disability [128]. It is therefore worth examining the potential impact of these parameters on improving quality of life in tDCS studies and creating homogeneous groups to exclude their variable impact on the simulation results.

#### 5.5.7. Level of Education

Several studies have shown that a higher level of education results in a higher quality of life in MS patients [103,126,129]. Higher-educated individuals feel more secure and have more opportunities to create and carry out their ideas. Furthermore, having this knowledge might help individuals evaluate their health, identify any anomalies more quickly, and start therapy sooner [102]. In studies on tDCS, it is therefore worth measuring the level of education, considering its impact on the quality of life in patients undergoing treatment, and creating homogeneous samples in terms of education level.

#### 5.5.8. Age

It is not obvious how age affects the quality of life of MS patients. According to some studies, quality of life declines with age, often because older people have had MS for a long time [115,130,131]. On the other hand, the research [102] revealed that the QoL in the PWB and COP subscales, which relate to mental health and coping with illness, increases with patient age. Similarly, other research [118,132] found a favorable relationship between age and mental health assessment. Individuals with long-term chronic illnesses must constantly adjust to changes in their condition brought on by both new and persistent symptoms that get worse over time. The most important thing in this situation is to identify the most effective coping mechanisms typically acquired over life. Older patients are more likely to assess their mental health as higher quality and to cope with the sickness better due to their well-developed adaptation mechanisms [102]. Therefore, although the impact of age on the quality of life in patients with MS is unclear, future studies should create homogeneous groups of patients in terms of age to avoid confounding related to the influence of this parameter on treatment outcomes.

### 5.6. Limited Understanding of Mechanisms

The processes by which tDCS affects the quality of life in MS patients are still poorly understood, despite conjecture regarding plausible modes of action, including reduction of beta-amyloid deposition, cerebral blood flow modulation, salience network engagement, and synaptic plasticity. To give a more thorough understanding of the effects of tDCS, future studies should attempt to clarify these pathways using neurophysiological, neuroimaging, and molecular studies. First of all, the potential of tDCS to increase cerebral blood flow should be further investigated in larger patient cohorts. Advanced imaging techniques, such as positron emission tomography (PET), or more cost-effective alternatives, such as near-infrared spectroscopy (NIRS), could be instrumental in conducting such investigations.

### 5.7. Taking into Account the Possible Influence of Skull Thickness on tDCS Results

Skull thickness exhibits significant variability influenced by factors such as sex, age, genetics, and population characteristics. Studies indicate that females often have slightly greater cranial vault thickness in specific regions, particularly the frontal and temporal bones [133]. These differences are primarily attributed to hormonal influences, such as estrogen’s role in preserving bone density [133]. However, these sex-based variations are not consistently observed across all populations and are often subtle.

Age-related changes in skull thickness follow distinct patterns. The cortical layers of the cranial vault, including the outer and inner tables, are generally thin with age, particularly in males. Between the ages of 20 and 100 years, cortical thinning can range from 36% to 60%, driven by reduced bone formation and increased resorption. In contrast, the diploic layer, the spongy bone between the cortical tables, often expands with age. This compensatory expansion can result in a net increase in overall skull thickness for some individuals, particularly in females. This age-related diploic layer expansion may counteract cortical thinning and help maintain skull integrity in older females, while males tend to show more uniform thinning across all layers [134].

Geographic and ancestral factors also play a significant role in skull thickness variation. For example, individuals from southern European populations generally have thicker cranial vaults compared to those from northern Europe, likely due to genetic adaptations to environmental pressures such as climate [135]. Genetic determinants of skull thickness have also been studied, with loci such as *WNT16* and *TNFSF11* implicated in influencing bone density. While these genes significantly impact postcranial bone density, their impact on cranial vault thickness is less clear [133]. Moderate heritability of skull morphology has been suggested, shaped by genetic predisposition and environmental factors [135].

Study [136] highlights the significant role of skull thickness in determining the distribution and strength of the electric field during tDCS, which directly affects its effectiveness. Regions with thinner skulls exhibit stronger electric fields, as the current encounters less resistance when passing through to the brain. Additionally, the composition of the bone significantly influences current distribution. Regions with a higher proportion of spongy bone, such as the parietal, frontal, and occipital areas, facilitate a more homogenous current distribution, while areas with more compact bone may restrict it. The distance between the scalp and cortical surface is another factor impacting current density, with increased distances reducing the amount of current reaching the brain. This space, often filled with cerebrospinal fluid (CSF), enhances conductivity but diminishes spatial resolution. Variability in skull morphology, including differences in thickness, the ratio of spongy to compact bone, and cortical gyrification, contributes to substantial inter-individual variability in tDCS effects. Computational modeling studies corroborate these findings, showing that thinner skull areas allow more current to penetrate the cortex, while thicker regions, depending on their composition, may alter current flow and distribution.

This information is particularly relevant for the design of tDCS studies in MS patients. Research has shown that individuals with MS frequently exhibit lower bone mineral density (BMD) compared to healthy controls. This reduction is typically more pronounced at the femoral neck than at the lumbar spine. Importantly, in ambulatory MS patients, BMD is reduced early in the disease process, even before significant disability develops. Factors such as immobility, muscle weakness, and inflammatory processes are believed to contribute to this decrease [137,138]. Although no studies have specifically examined the skull bones in MS patients, these findings can be cautiously extrapolated to cranial bones.

Future tDCS studies in MS patients should take into account skull thickness as a key parameter and investigate its impact on treatment outcomes, not only regarding quality of life but also in relation to other symptoms and clinical aspects. Skull thickness measurements should be conducted for all participants, and study samples should be divided into groups with similar skull characteristics. This approach will minimize patient heterogeneity and enhance the reliability and validity of the results.

### 5.8. Integrating Artificial Intelligence to Enhance tDCS in MS Care

Emerging advancements in artificial intelligence (AI) offer an innovative horizon for optimizing tDCS protocols in complex neurological conditions like multiple sclerosis. As explored by Rudroff et al. [139], AI-guided approaches can personalize tDCS parameters by integrating extensive datasets, including clinical profiles, neuroimaging, and real-time physiological data. These methods promise to enhance treatment specificity and adaptability, addressing inter-patient variability and fluctuating symptoms often observed in MS. Specifically, AI could aid in tailoring tDCS protocols to individual symptom profiles and underlying pathophysiological mechanisms, dynamically adjusting stimulation parameters based on ongoing feedback from wearable sensors and neuroimaging, and developing predictive models to stratify patients most likely to benefit from tDCS. Furthermore, AI offers opportunities to optimize clinical trial designs and reduce heterogeneity in study populations while providing insights into neural connectivity patterns linked to symptom improvements, thereby enhancing our understanding of tDCS mechanisms in MS. By facilitating the global application of tDCS technologies through AI-driven frameworks, these advancements can address challenges such as data privacy and equitable access. This AI-tDCS synergy underscores the potential to move beyond one-size-fits-all paradigms, enabling transformative, precision-based neuromodulation therapies that could substantially improve the quality of life in MS patients. Future research should focus on clinical validation of these approaches, ensuring robust, ethical, and globally implementable solutions.

## 6. Conclusions

Quality of life is an essential parameter in assessing the functioning of patients with MS. Ensuring the best possible quality of life should be a priority in the clinical care of this population. Currently used psychological interventions are effective in this field, but the need for knowledge about the mechanisms of action and the small amount of research result in the need to develop new therapeutic methods. One such was tDCS, which was tested in this context. Four of the seven studies included in this review showed improvements, of which all (two) improved after anodal stimulation of the left DLPFC. Stimulation of the motor cortex has shown mixed results. For this promising result (prefrontal stimulation), the use of prefrontal tDCS can be useful to improve the quality of life in patients with MS. Further research is required to confirm these results. The positive results encouraged attempts to elucidate potential mechanisms of action. These include improved synaptic plasticity, improved cerebral blood flow, reduction of beta-amyloid accumulation, and salience network engagement through tDCS. The discussion section also highlighted the impact of various factors on quality of life and recommended that future studies should measure and control them to exclude confounding results.

## Figures and Tables

**Table 2:** MSQOL54 quality of life scores in included studies by subscales.

Additional Information	MSQOL54 Mental After tDCS	MSQOL54 Physical After tDCS	MSQOL54 Mental Before tDCS	MSQOL54 Physical Before tDCS	Citation
The results before and after treatment were presented as a whole, without being separated into individual subscales.	x	x	x	x	[68]
The results before and after treatment were presented as a whole, without being separated into individual subscales.	x	x	x	x	[69]
x	70.2	52.5	68.3	47.9	[70]
x	After tDCS: 45.72 ± 3.2	After tDCS: 53.99 ± 2.9	44.40 ± 2.90	50.16 ± 2.20	[72]
	Follow-up (4 weeks): 44.84 ± 3.0	Follow-up (4 weeks): 50.00 ± 1.4			
The study did not provide any numerical data.	x	x	x	x	[73]
The results before and after treatment were presented as a whole, without being separated into individual subscales.	x	x	x	x	[74]

“x” means “no applicable”.

## Data Availability

No new data were created or analyzed in this study. Data sharing is not applicable to this article.
